# Recurrent, sequential, bilateral deep cerebellar hemorrhages: a case report

**DOI:** 10.1186/1752-1947-5-360

**Published:** 2011-08-10

**Authors:** Osama SM Amin, Raz T Omer, Aso A Abdulla, Raz H Ahmed, Omed Ahmad, Soran Ahmad

**Affiliations:** 1 Department of Neurology, Sulaimaniya General Teaching Hospital, Sulaimaniya City, Iraq; 2 Department of Medicine, Sulaimaniya General Teaching Hospital, Sulaimaniya City, Iraq

## Abstract

**Introduction:**

Hypertensive intra-cerebral hemorrhage is usually a one-time event and recurrences are rare. Most recurrences develop as part of long-term failure of blood pressure control. The site of the re-bleed is usually limited to the basal ganglia and thalami.

**Case presentation:**

We report the case of a 59-year-old hypertensive Caucasian woman who developed two sequential, right- and then left-sided, deep cerebellar hemorrhages. The second hemorrhage followed the first one by 57 days, at a time when her blood pressure was optimally controlled. In spite of these critical sites and short duration between the two bleeds, the patient achieved a relatively good functional recovery. Her brain magnetic resonance angiogram was unremarkable.

**Conclusion:**

The development of recurrent hypertensive hemorrhage is rare and usually occurs within two years of the first bleed. To the best of our knowledge, this is the first reported case of bilateral, sequential, right- and then left-sided deep cerebellar hemorrhages. These hemorrhages were separated by eight weeks and the patient had a relatively good functional recovery. We believe that hypertension was the etiology behind these hemorrhages.

## Introduction

Hypertensive intra-cerebral hemorrhage is usually a one-time event and recurrences are rare. Most of these recurrences develop as part of a failure of blood pressure control and within two years of the first hemorrhage. The sites of the re-bleed are usually limited to the basal ganglia and thalami.

## Case presentation

**Figure 1:**
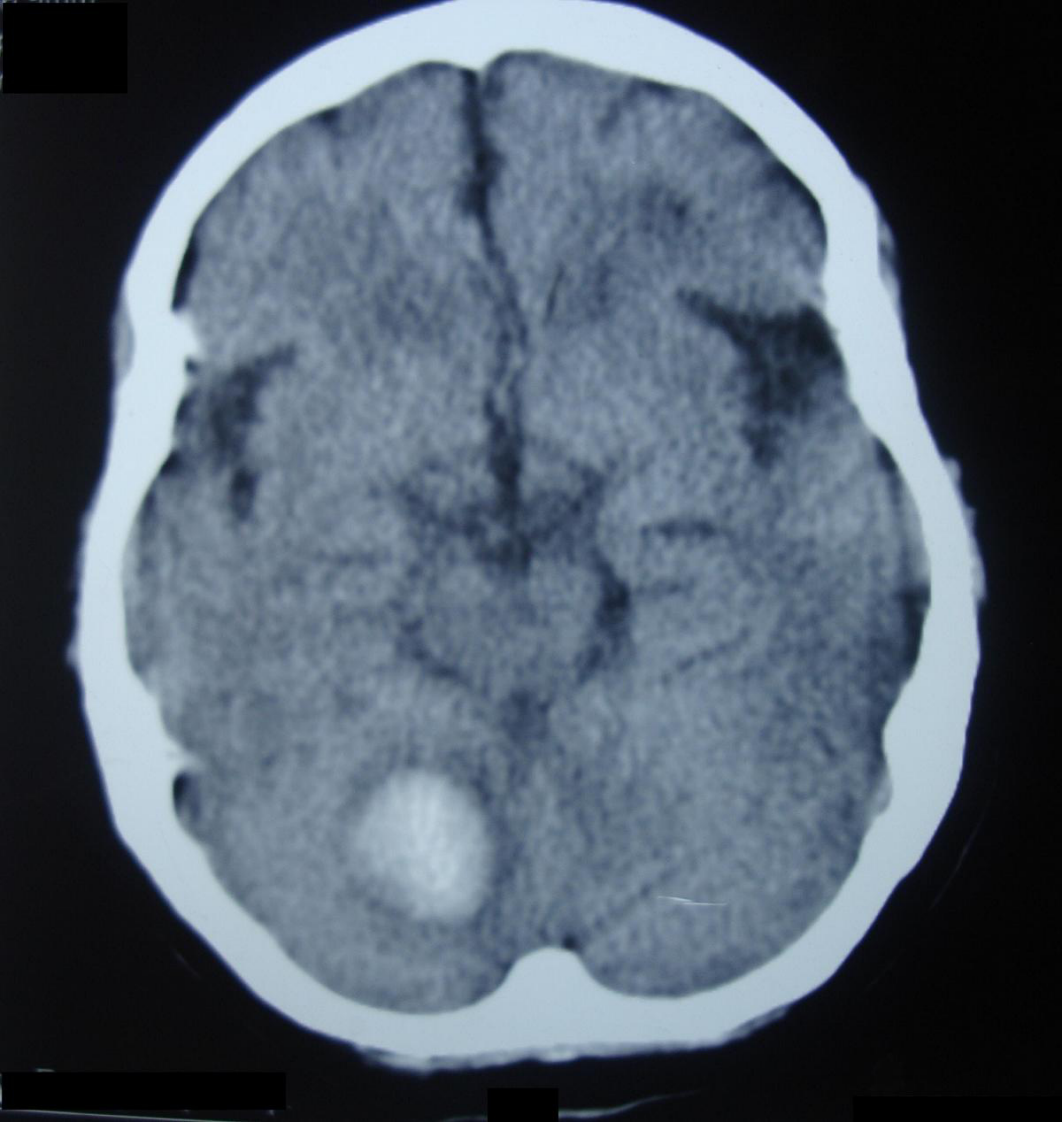
**Non-contrast brain CT scan of the patient, which was done approximately four hours after developing her symptoms**. Note that the hematoma lies at the deep right cerebellar hemisphere and is surrounded by mild cytotoxic edema. The fourth ventricle was not compressed and brainstem signs were absent.

A 59-year-old Caucasian woman presented with severe headache, repeated vomiting, and instability of stance and gait to our Accident and Emergency (A&E) department. She had been experiencing these symptoms for three hours. The patient had long-standing poorly-controlled essential hypertension, for which she took oral atenolol. The family denied head trauma or the ingestion of other medications. She was drowsy and had a blood pressure of 210/130 mmHg and a pulse rate of 110 beats per minute. Her lab tests (which included a coagulation screen) were unremarkable but her emergency non-contrast brain computed tomography (CT) scan revealed right-sided acute deep cerebellar hematoma with mild surrounding edema; no ventricular dilatation developed (Figure 1). She was managed as a case of primary spontaneous hypertensive intra-cerebral hemorrhage. During the following two weeks, she showed a favorable improvement and then she was discharged home on enalapril, metoprolol, hydrochlorothiazide, and simvastatin. Her blood pressure was 125/75 mmHg at that time. She was able to stand and walk with some assistance and her speech was normal. Two weeks later, the patient came in for a scheduled follow-up visit. She was conscious and her speech was normal; she could stand and walk alone, and her blood pressure was 110/85 mmHg.

**Figure 2:**
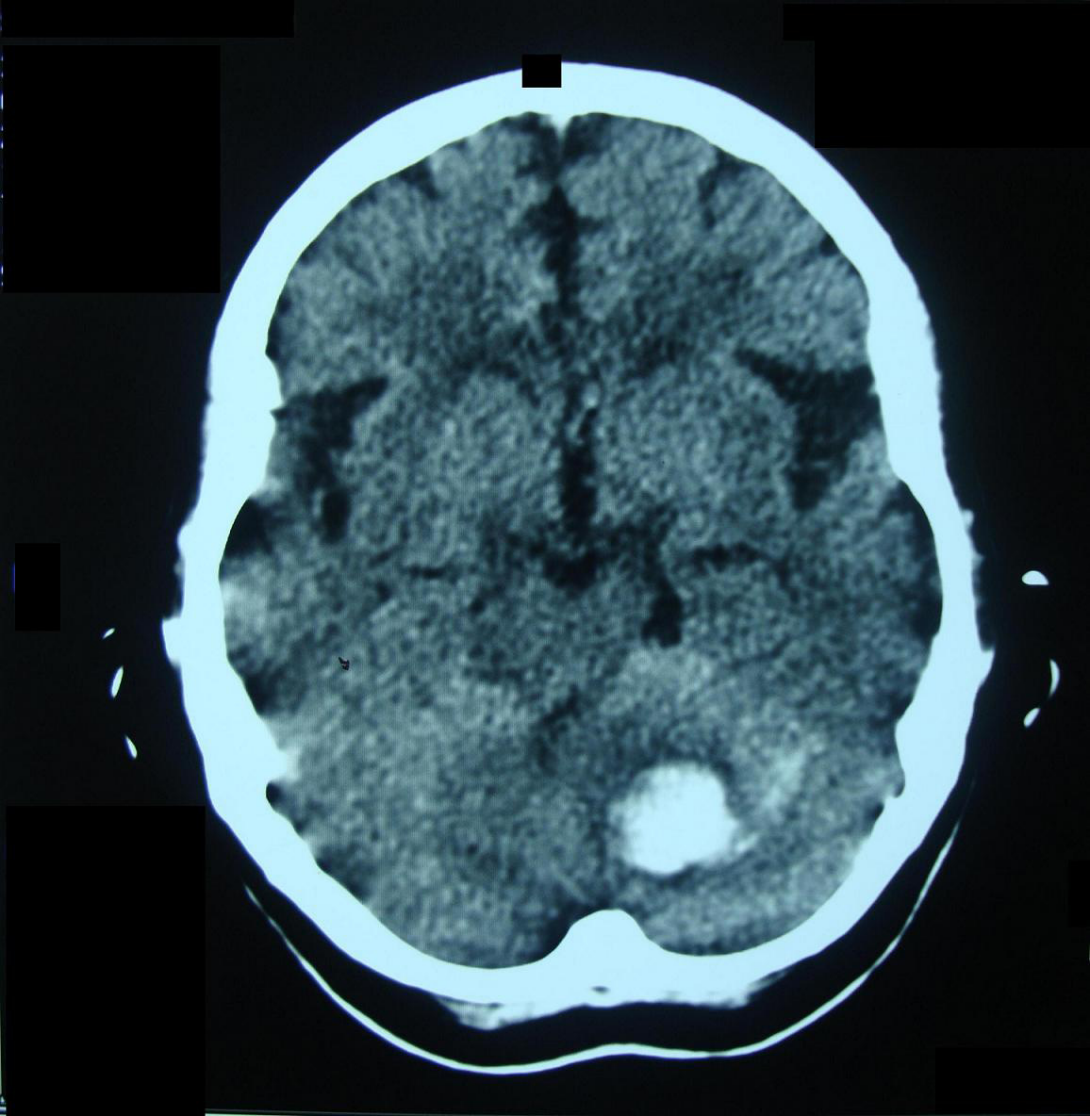
**Non-contrast brain CT scan of the same patient, which was done about 2 hours after the re-bleed**. The second hemorrhage lies at the left deep dentate nuclei and is not surrounded by edema, implying a very recent development. The first hematoma at the right cerebellar hemisphere had resolved, leaving a small slit.

Eight weeks later, the patient presented with drowsiness, slurring of speech, vomiting, and inability to sit and stand unaided for one hour to our A & E. Her blood pressure was 190/100 mmHg. Her routine blood tests were within their normal reference range. An emergency non-contrast brain CT scan showed left-sided acute deep cerebellar hematoma, a contralateral site to the first hematoma (Figure 2). The patient was treated medically and improved gradually over a two week period. On discharge, her speech was scanning and her gait was wide-based and ataxic. She could stand and walk alone with minor assistance. Because of the lack of expertise in our radiology department, conventional cerebral angiography was not ordered; however, a brain magnetic resonance angiogram (MRA) was done two weeks later and the result was unremarkable. We assume that our patient's hemorrhages were hypertensive in etiology.

## Discussion

Mohr *et al*. [1] analyzed 694 hospitalized stroke patients and found that intra-cerebral hemorrhage (ICH) is the third most common cause of stroke; embolic ischemic stroke and atherothrombotic infarction ranked second and first on that list of frequency, respectively. ICH constitutes 10% to 15% of all stroke subtypes [2].

Long-standing arterial hypertension is responsible for about 50% of all cases of primary ICH [3], and according to Thrift and colleagues [4] this hypertension doubles the risk of developing ICH. The necrotizing effect of long-standing hypertension on the wall of small penetrating blood vessels (<300 μm in diameter) leads to the formation of Charcot-Bouchard micro-aneurysms; rupture of the latter leads to intra-parenchymal hemorrhage [5]. Approximately 20% of hypertensive hemorrhages develop in the posterior fossa; the rest are supratentorially located. In the cerebellum, the small penetrating branches of superior cerebellar arteries (and posterior inferior cerebellar arteries to a lesser extent) on either side are the usual target for micro-aneurysmal formation [6-8]. Therefore, most hemorrhages appear in the region of the dentate nuclei. These cerebellar hemorrhages account for approximately 5% to 15% of all primary ICHs [9-12]; the cerebellum is the fourth most common site for spontaneous ICHs, trailing thalamic, lobar, and putamenal hemorrhages [13].

In 1984, Kunitz and coworkers [14] respectively analyzed the NINCDS Stroke Data Bank. Only one out of 101 patients with hemorrhagic strokes had a history of intra-cerebral hemorrhage. Therefore, primary spontaneous ICH can be considered a one-time event. Douglas and Haerer [15] found that hypertensive intra-cerebral hemorrhages, unlike Berry's aneurysms, rarely, if ever, re-bleed at the same site. On the other hand, patients are not likely to have a second bleed in another location. According to Gonzalez-Duarte and colleagues [16], recurrent hypertensive intra-cerebral hemorrhages do occur, but at a very low rate, and the main topographic pattern of re-bleeding is basal ganglionic-ganglionic. Non-hypertensive recurrent hemorrhages tend to be lobar in location, in contrast to the hypertensive ones. Bae and associates [17] concluded that the recurrence rate is 5.4% and that most recurrences develop within two years of the first hemorrhage. They also found that all of these re-bleedings occurred at sites different from the first ones, but the majority were within the basal ganglia and thalami and all were related to poor arterial blood pressure control.

The short-term mortality of recurrent hypertensive hemorrhages is considerably higher (32%) [16] than that of the first hemorrhages (20%) [18]. As for the long term functional outcome, Portenoy *et al*. [19] found that 55% of patients achieve a good functional recovery after sustaining a hypertensive ICH, a figure that falls to 23% if a recurrence develops [16].

Our patient developed a right-sided deep cerebellar hemorrhage; the subsequent eight weeks were marked by a good functional recovery and optimal blood pressure control. Another hemorrhage at the left dentate nuclei occurred after 57 days and resolved in a relatively favorable functional independence. Conventional cerebral angiography was not done because of the lack of expertise in our hospital's radiology department. Brain MRA after two weeks revealed no vascular anomaly.

Although hypertension is the most common etiology behind the development of non-traumatic intra-cerebral hemorrhage in adults [3,20,21], the occurrence of recurrent hemorrhages should always prompt the physician to search for an underlying cause(s), such as multiple ischemic strokes with secondary hemorrhagic transformation, reperfusion after thrombolytic therapy, extension from a subarachnoid bleed, vascular anomalies, tumors, congophilic angiopathy, blood dyscrasias, vasculitis, coagulopathy, and illicit drug use [22-27]. Our patient's clinical features, examination, and work-up have excluded the risk factors listed above.

## Conclusion

The development of recurrent hypertensive hemorrhage is rare and usually occurs within two years of the first bleed. To the best of our knowledge, this is the first reported case of bilateral, sequential, right- and then left-sided, deep cerebellar hemorrhage. The hemorrhages occurred eight weeks apart and she had a relatively good functional recovery. We believe these hemorrhages were hypertensive in etiology.

## Consent

Written informed consent was obtained from the patient for publication of this case report and any accompanying images. A copy of the written consent is available for review by the Editor-in-Chief of this journal.

## Competing interests

The authors declare that they have no competing interests.

## Authors' contributions

Clinical management and follow-up was made by OSMA. OA, SA, RHA, RTO, and AAA examined the patient, took the photos of the brain imaging and performed the photo editing process. The literature search was done by OSMA. OSMA wrote the manuscript, and all authors read and approved its final draft.
